# Tension‐Reducing Arthroscopic Rotator Cuff Repair Using High‐Strength Suture Tape in a Suspension Bridge Configuration

**DOI:** 10.1002/atn2.70335

**Published:** 2026-10-29

**Authors:** Shunnan Liu, Hongbo Wen, Zhe Dou, Yizhong Ren, Xiping Feng, Yuyan Na

**Affiliations:** ^1^ Department of Sports Medicine The Second Affiliated Hospital of Inner Mongolia Medical University Hohhot Inner Mongolia Autonomous Region China; ^2^ Inner Mongolia Medical University Hohhot Inner Mongolia Autonomous Region China

## Abstract

The maturation of arthroscopic techniques has provided patients with rotator cuff tears a minimally invasive treatment option with favorable outcomes; however, the rate of retear remains high. High‐strength suture tape is widely used in procedures such as knee ligament reconstruction and ankle ligament repair, where it enhances the tensile strength during joint motion, thereby reducing the risk of ligament rerupture. In this report, we describe an arthroscopic surgical technique for rotator cuff repair using high‐strength suture tape for tension relief. The key steps include passing the high‐strength suture tape in a “U‐shaped” configuration through the tendon stump, reducing the tendon by securing it with a lateral‐row anchor, and then, under nearly “zero tension,” using the suture tails from the medial‐row anchors to approximate the remaining tendon stump. This technique, by employing high‐strength suture tape to reduce the tendon and share the tension load, significantly minimizes the cutting effect of the medial‐row anchors on the tendon, thereby lowering the risk of tendon retear and promoting tissue healing.

## Graphical abstract

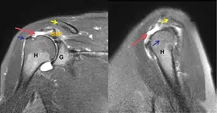

**VIDEO 1:** Arthroscopic rotator cuff repair using high‐strength suture tape for tension relief and a “zero‐tension” suture technique with medial‐row anchors. The arthroscopic view reveals a crescent‐shaped, full‐thickness rotator cuff tear. After assessing the tear angle and retraction direction to confirm repairability, the adhesions around the tendon are released, the tendon stump is debrided, and the footprint is freshened. A high‐strength suture tape is passed through the tendon stump in a “U‐shaped” configuration and set aside. Two medial‐row anchors are then inserted into the footprint, with 1 limb of their sutures passed through the tendon stump and set aside. The lateral‐row anchor is inserted in conjunction with the suture tape to reduce the tendon and is secured just inferior to the greater tuberosity. At this point, the tendon is reduced, and the tear gap is closed. Finally, the sutures from the medial‐row anchors are tied under nearly “zero tension” to approximate the remaining tendon stump. This technique is designed to use the high‐strength suture tape for load sharing, thereby reducing the cutting effect of the sutures on the tendon during repair and subsequently lowering the risk of retear. Video content can be viewed at https://doi.org/10.1002/atn2.70335.

With the popularization of national fitness, rotator cuff tears have a prevalence rate of nearly 50% in adults over 50 years old. Although conservative treatment may yield comparable clinical outcomes for patients over 55 years old with small, nontraumatic supraspinatus tendon tears,^1^ its success rate decreases with the severity of the tear.^2^ For the management of supraspinatus tendon tears, arthroscopic minimally invasive surgery is the mainstay clinical approach to restore the anatomical structure and biomechanical function of the rotator cuff tissue. Owing to its advantages of minimal trauma and rapid recovery, it has become the first‐line treatment for rotator cuff tears.^3^, ^4^

In the surgical treatment process, apart from the tendon coverage of the footprint, the cutting force exerted by anchor sutures on the tendon and the tension required for reconstructing the retracted tendon are important factors contributing to postoperative retearing. Studies have indicated that intraoperative repair tension reaching or exceeding 10 N significantly increases the risk of retearing.^5^ Despite the continuous advancement of arthroscopic repair techniques, existing single‐row or traditional double‐row techniques still have inherent deficiencies in footprint compression and biological healing, leading to a high rate of postoperative retearing.^6^ Therefore, a repair technique that achieves low‐tension suturing while enhancing footprint coverage has become an urgent clinical need to improve the healing rate of the supraspinatus tendon and reduce the risk of retearing.^7^, ^8^

The purpose of this technical note is to describe a supraspinatus tendon suture technique based on the core principle of “tension reduction with suture tape and external row anchors plus tension‐free suture with internal row anchors,” as well as its mechanism, technical key points, and application prospects.

## SURGICAL TECHNIQUE

### Indications

Indications for this technique include full‐thickness supraspinatus tendon tears with moderate to severe retraction, in which conventional repair would require excessive tension. Contraindications include irreparable massive tears, severe osteoporosis with the high risk of anchor pullout, and active infection. Preoperative magnetic resonance imaging examination is of great significance for the diagnosis of rotator cuff injuries. In this case, preoperative magnetic resonance imaging showed an obvious full‐thickness tear of the supraspinatus tendon (Figure 1).

**FIGURE 1:**
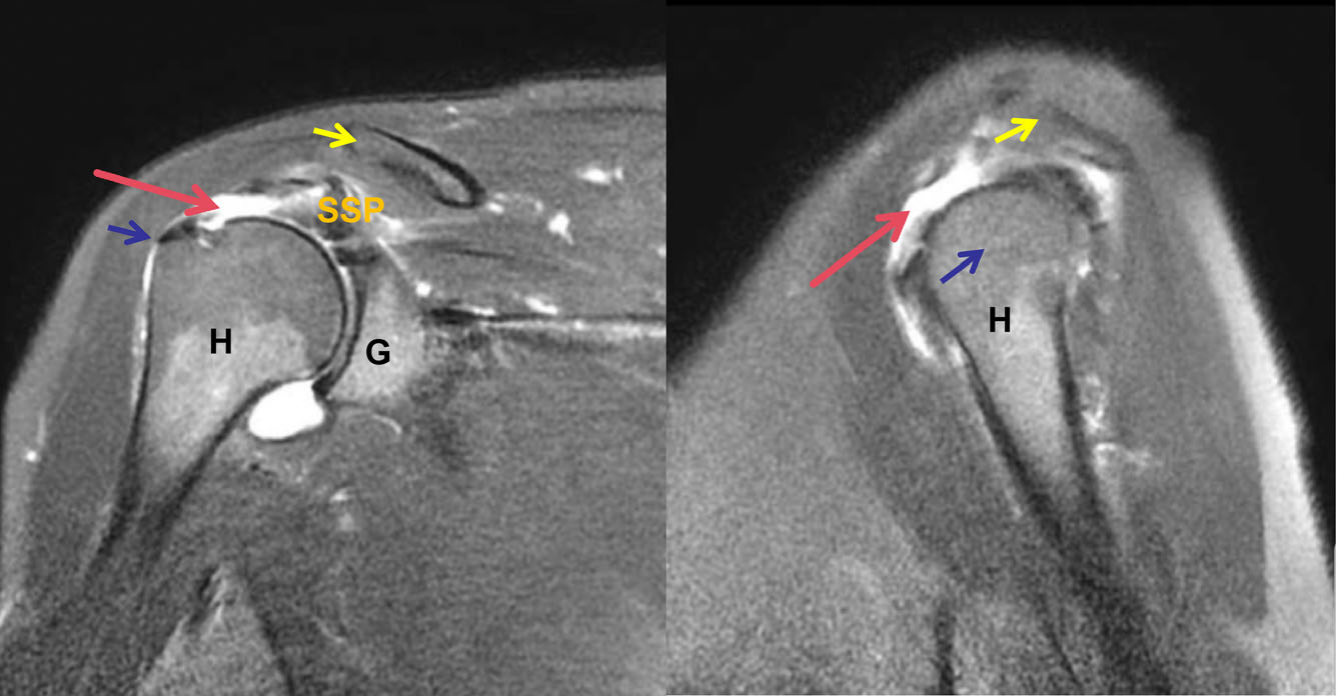
Preoperative MRI in supine position. Left: coronal MRI image; right: sagittal MRI image. The red arrow indicates the complete full‐thickness tear of the supraspinatus tendon with obvious retraction of the tendon stump. The yellow arrow indicates the acromion. The blue arrow indicates the greater tuberosity of the humerus. (G, glenoid; H, humerus; MRI, magnetic resonance imaging; SSP, supraspinatus.)

### Patient Positioning

General anesthesia is administered. After confirming a satisfactory anesthetic effect, the patient is placed in the lateral decubitus position using an inflatable positioner. The affected upper limb is distracted with a traction device at a 30° to 45° superolateral angle.

### Portal Establishment and Visualization

The operative area is routinely disinfected and draped. A posterolateral portal is established, and the arthroscope is introduced into the glenohumeral joint for diagnostic inspection. An anteroinferior portal is then created. The inflamed synovium is debrided using a shaver and a radiofrequency ablator. Associated structures, including the long head of the biceps tendon, the glenoid labrum, and the subscapularis tendon, are evaluated and addressed appropriately. The arthroscope is subsequently advanced into the subacromial space. The inflamed subacromial bursa is debrided with a shaver and radiofrequency ablator to achieve a clear operative field. The acromion is assessed; in the presence of subacromial impingement, a burr is used to resect any osteophytes. After meticulous hemostasis, an adequate working space is confirmed. A full‐thickness, crescent‐shaped tear of the supraspinatus tendon with retraction is visualized (Figure 2).

**FIGURE 2:**
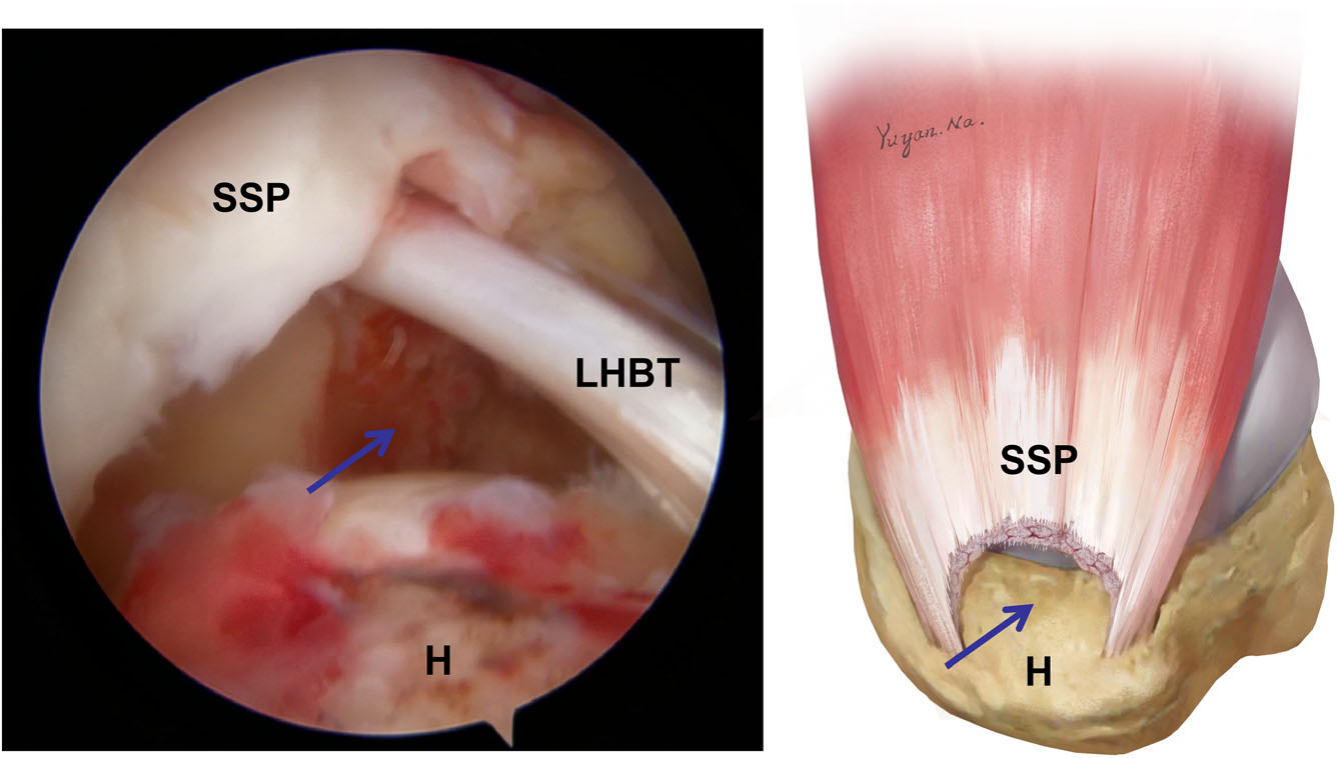
Left: arthroscopic view through an inferolateral acromial portal into the subacromial space. The blue arrow indicates a full‐thickness tear of the supraspinatus tendon, which appears crescent‐shaped, with obvious retraction of the tendon stump. Right: corresponding schematic diagram. (H, humerus; LHBT, long head of the biceps tendon; SSP, supraspinatus.)

### Tendon Preparation and Preliminary Reduction

The edges of the torn supraspinatus tendon are refreshed and debrided using a shaver. A tissue grasper is used to mobilize the supraspinatus tendon back toward its footprint on the greater tuberosity, allowing assessment of the tear and retraction pattern. A preliminary attempt at tendon reduction reveals substantial tension.

### High‐Strength Suture Tape Passage

The remnant of the supraspinatus tendon is evenly divided into anterior, middle, and posterior zones. A suture passer loaded with a polydioxanone suture is used to shuttle 1 limb of a high‐strength suture tape through the tendon remnant between the anterior and middle zones via the anteroinferior portal. The same maneuver is repeated through a posterior portal to pass the other limb of the suture tape between the middle and posterior zones. The suture tape is now positioned in a U‐shaped configuration through the tendon stump (Figure 3).

**FIGURE 3:**
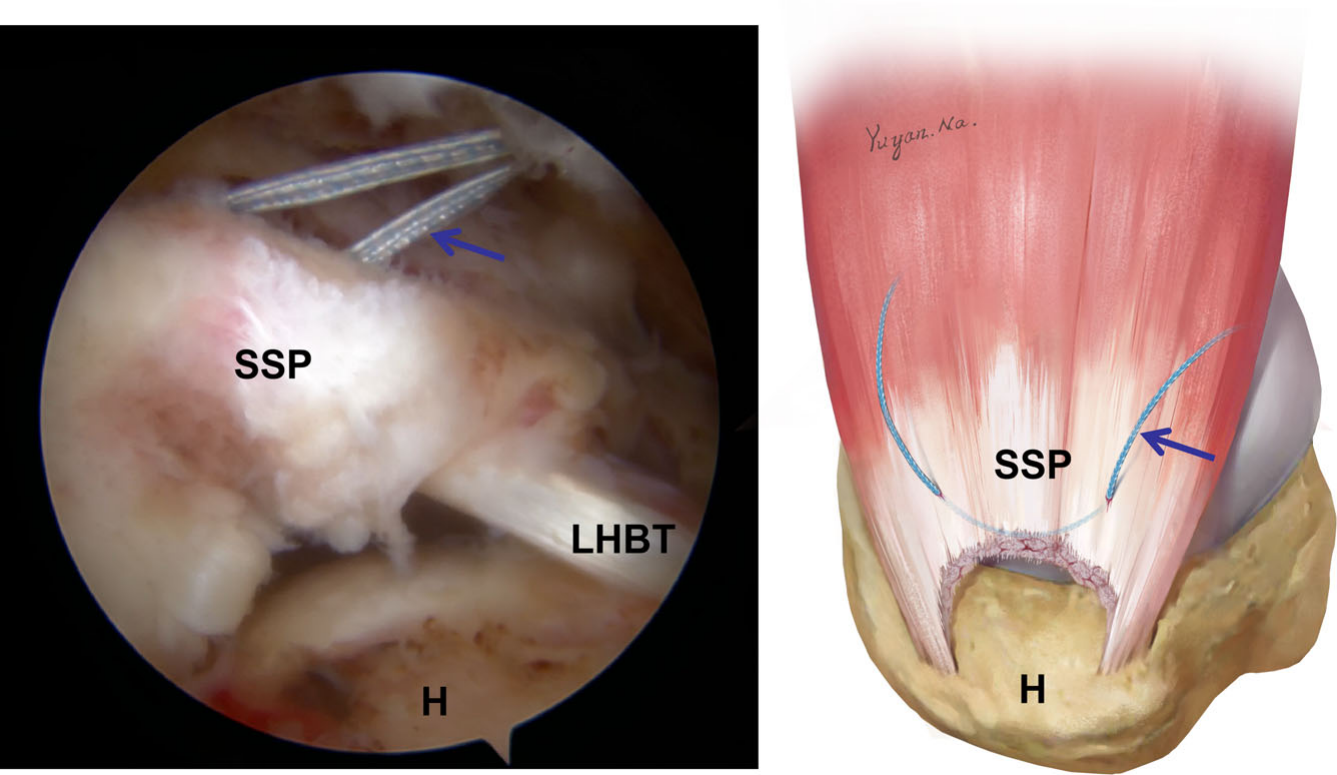
Left: arthroscopic view. The blue arrow shows the suture tape passed through the supraspinatus tendon stump in a U‐shaped configuration and pulled upward in an attempt to reduce the tendon. Right: corresponding schematic diagram. (H, humerus; LHBT, long head of the biceps tendon; SSP, supraspinatus.)

### Medial‐Row Anchor Placement

The greater tuberosity footprint is debrided and lightly decorticated with a burr to create a bleeding bed. Two pilot holes are created, and a medial‐row anchor (4.5‐mm SwrilFix PK; Double Medical Technology, Xiamen, China) is inserted at the corresponding location on the tendon footprint. The sutures from the medial anchor are sequentially passed through the anterior, middle, and posterior zones of the tendon using a suture passer and polydioxanone suture and are set aside for later use.

### Tendon Reduction and Lateral‐Row Fixation

Both limbs of the previously placed suture tape are loaded into a lateral‐row anchor (5.0 mm SwirlLock PK; Double Medical Technology). Traction is applied to the suture tape in line with the direction of tendon reduction. The tendon is successfully reduced to its footprint with a good approximation of the tear edges and appropriate tension. While maintaining the reduction, the lateral‐row anchor is impacted and secured distal and lateral to the greater tuberosity of the humerus. Satisfactory reduction of the torn supraspinatus tendon is confirmed (Figure 4). Notably, the suture tape exhibits a markedly reduced tendency to cheese‐wire through the tendon tissue compared with standard sutures from the medial anchor.

**FIGURE 4:**
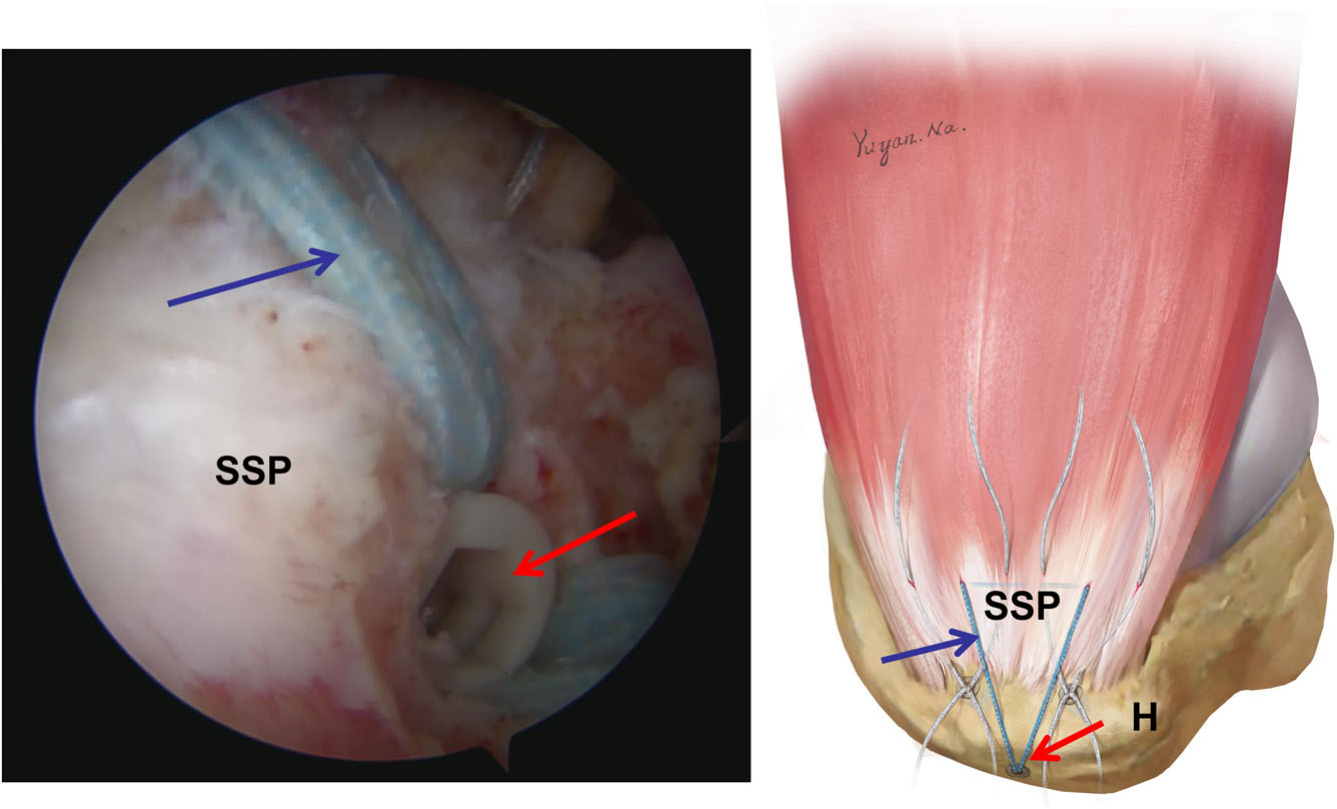
Left: arthroscopic view. The blue arrow shows the tail of the suture tape passing through the lateral‐row anchor and fixed to the lateral aspect of the greater tuberosity (red arrow). The supraspinatus tendon stump has been almost completely reduced. Right: corresponding schematic diagram. (H, humerus; SSP, supraspinatus.)

### Completion of Tendon Repair

Exploration of the remaining anterior and posterior tear zones reveals significantly reduced tension and good closure of the tear margins. The sutures from the medial‐row anchor are then tied sequentially under near‐zero tension, completing the repair of the remaining tendon. The final arthroscopic evaluation confirms excellent reduction and secure fixation of the supraspinatus tendon (Figure 5), concluding the procedure. The postoperative magnetic resonance imaging results of the affected shoulder are shown in Figure 6.

**FIGURE 5:**
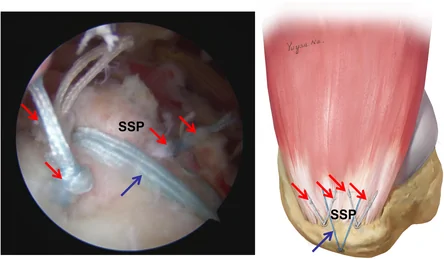
Left: arthroscopic view. The 4 red arrows indicate the knot of the medial‐row anchor sutures. The blue arrow shows the suture tape retracting and reducing the supraspinatus tendon stump. The supraspinatus tendon is completely reduced and apposed to the footprint. The suture tape and sutures have appropriate tension without obvious cut‐through. Right: corresponding schematic diagram. (SSP, supraspinatus.)

**FIGURE 6:**
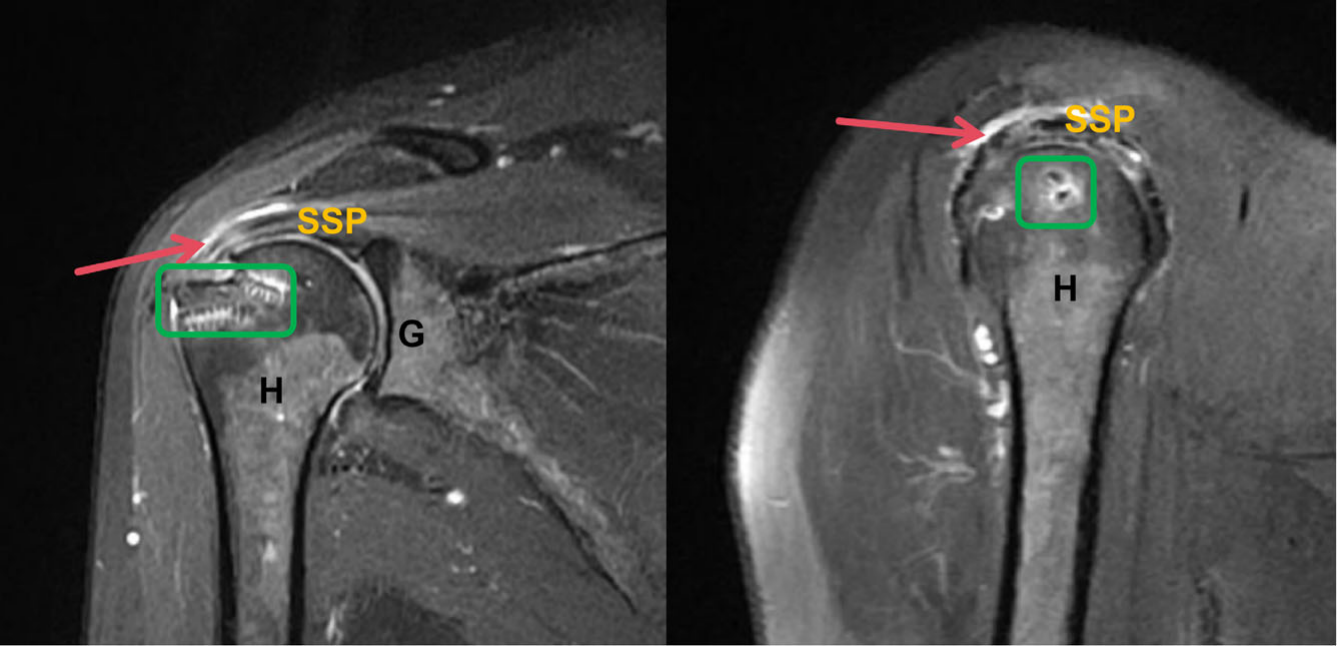
Postoperative MRI in supine position. Left: coronal MRI image; right: sagittal MRI image. The red arrow indicates the postoperative appearance of the repaired supraspinatus tendon, showing good continuity and complete apposition to the footprint. The green box indicates the location of the anchors. (G, glenoid; H, humerus; MRI, magnetic resonance imaging; SSP, supraspinatus.)

The detailed surgical procedure can be referred to in the attachment (Video 1). The advantages and limitations are shown in Table 1. The pearls and pitfalls are shown in Table 2.

**TABLE 1:** Advantages and Limitations of the Bridge Tension‐Reducing Technique

**Advantages**	**Limitations**
1. The suture tape, being wider and stronger than traditional sutures, exerts a minimal cutting effect on the tissue when pulling and reducing the tendon	1. Increased operation time and the technical difficulty for the operator
2. After reducing the tendon using the suture tape and lateral‐row anchors, the medial‐row anchor tails can be tied with almost zero tension	
3. These tapes have greater resistance to creep compared with autologous tendons, fascia lata, or acellular dermal matrix	2. Compared with the traditional double‐row anchor repair technique, this method results in a smaller contact area between the tendon and the footprint
4. The U‐shaped downward pulling and reduction of the tendon with the suture tape result in a larger tendon‐to‐footprint contact area compared with traditional single‐row repair, making it more consistent with the original anatomy	

**TABLE 2:** Pearls and Pitfalls of the Bridge Tension‐Reducing Technique

**Pearls**	**Pitfalls**
1. Both ends of the suture tape must pass through the anterior and posterior one‐third of the tendon stump to ensure uniform stress distribution during traction	1. When the tape is tensioned to reduce the tendon, stress is concentrated at the tape‐tendon interface; if the tissue quality of the tendon stump is poor, there is a risk of tendon retear
2. The patient should be placed in a lateral position with the affected shoulder abducted at 30°‐45°. This position shortens the distance between the humeral greater tuberosity and the tendon stump, facilitating tendon reduction	2. The risk of anchor pullout should be closely monitored in patients with osteoporosis
3. The direction of tendon retraction should be assessed preoperatively to determine the optimal traction direction for reduction, aiming to achieve anatomical reduction as closely as possible	

## DISCUSSION

The key to successful surgical treatment of supraspinatus tendon tears lies in achieving anatomical reduction as much as possible, maximizing tendon‐bone contact area, and promoting subsequent healing.^9^ However, in clinical practice, traditional single‐row or double‐row suture techniques alone often fail to achieve satisfactory clinical outcomes because of tendon retraction, degeneration, and excessive reduction tension.^10^, ^11^ Therefore, the development of rotator cuff repair techniques is essential to improve surgical prognosis and reduce the risk of retearing.

From a biomechanical perspective, this technique establishes robust mechanical support through the external row anchors and suture tape acting as a tension bridge. It disperses repair tension over the wider surface of the suture tape and ultimately achieves tension‐free fixation with the internal row anchors. This collaborative mechanism of “external anchors bearing tension, internal anchors performing tension‐free repair and fixation” can further improve the initial stability of tendon repair, avoid suture cutting, reduce local compressive stress, and provide favorable biomechanical conditions for tendon‐bone healing. The “suture tape U‐reduction and zero‐tension suture repair technique for tendon tears” achieves a balance between anatomical reduction and mechanical stability through the innovative application of repair materials and a surgical approach, minimizing the risk of retearing caused by suture cutting. In clinical practice, this technique features clear operative steps and high efficacy, making it suitable for patients with full‐thickness supraspinatus tendon tears and high reduction tension. However, this technique also prolongs the operative time and requires a high level of surgical proficiency from the operator. Excessive or insufficient tension applied by the suture tape during tendon reduction can affect surgical outcomes. Additionally, clinicians should be alert to the risk of anchor pullout in patients with osteoporosis. Overall, this surgical technique has good learnability and great potential for clinical application and popularization. Future multicenter, large‐sample clinical studies are warranted to further verify its long‐term postoperative functional scores, healing rates, and complication rates, thereby providing stronger evidence‐based support for clinical practice.

## DISCLOSURES

The authors (S.L., H.W., Z.D., Y.R., X.F., Y.N.) declare that they have no known competing financial interests or personal relationships that could have appeared to influence the work reported in this article.

## FUNDING

This work was supported by the Natural Science Foundation of Inner Mongolia Autonomous Region (2024MS08008); the High‐Level Clinical Specialist Development and Technology Program for Public Hospitals of Inner Mongolia Autonomous Region (2024SGGZ089); the Science and Technology Program of Inner Mongolia Autonomous Region (2025YFSH0112); and the Joint Scientific Research Fund Technology Project of Public Hospitals (2025GLLH0223).
